# Drivers of the extreme North Atlantic marine heatwave during 2023

**DOI:** 10.1038/s41586-025-08903-5

**Published:** 2025-06-04

**Authors:** Matthew H. England, Zhi Li, Maurice F. Huguenin, Andrew E. Kiss, Alex Sen Gupta, Ryan M. Holmes, Stefan Rahmstorf

**Affiliations:** 1 Centre for Marine Science and Innovation (CMSI) and ARC Australian Centre for Excellence in Antarctic Science, University of New South Wales, Sydney, New South Wales Australia; 2 Research School of Earth Sciences and ARC Centre of Excellence for Climate Extremes, Australian National University, Canberra, Australian Capital Territory Australia; 3 Climate Change Research Centre (CCRC) and ARC Australian Centre for Excellence in Antarctic Science, University of New South Wales, Sydney, New South Wales Australia; 4 Australian Bureau of Meteorology, Sydney, New South Wales Australia; 5 Potsdam Institute for Climate Impact Research, Potsdam, Germany

**Keywords:** Physical oceanography, Attribution

## Abstract

North Atlantic Ocean circulation and temperature patterns profoundly influence global and regional climate across all timescales, from synoptic^1^ to seasonal^2–4^, decadal^5^, multidecadal^6,7^ and beyond^8,9^. During 2023, an extreme and near-basin-scale marine heatwave developed during Northern Hemisphere summer, peaking in July. The warming spread across virtually all regions of the North Atlantic, including the subpolar ocean, where a cooling trend over the past 50–100 years has been linked to a slowdown in the meridional overturning circulation^10,11^. Yet the mechanisms that led to this exceptional surface ocean warming remain unclear. Here we use observationally constrained atmospheric reanalyses alongside ocean observations and model simulations to show that air–sea heat fluxes acting on an extremely shallow surface mixed layer, rather than anomalous ocean heat transport, were responsible for this extreme ocean warming event. The dominant driver is shown to be anomalously weak winds leading to strongly shoaling (shallowing) mixed layers, resulting in a rapid temperature increase in a shallow surface layer of the North Atlantic. Furthermore, solar radiation anomalies made regional-scale warming contributions in locations that approximately correspond to some of the region’s main shipping lanes, suggesting that reduced sulfate emissions could also have played a localized role. With a trend towards shallower mixed layers observed over recent decades, and projections that this will continue into the future, the severity of North Atlantic marine heatwaves is set to worsen.

## Abstract (web summary)

Atmospheric reanalyses combined with ocean observations and model simulations show that the extreme 2023 North Atlantic heatwave was primarily driven by anomalously weak winds leading to strongly shoaling mixed layers, with a smaller contribution from clearer skies.

## Main

Marine heatwaves are characterized by sustained periods of abnormally warm ocean temperatures in a given region^12,13^, with the potential for severe damage to marine ecosystems^14^ and reliant human systems^15^. Marine heatwaves can also influence atmospheric conditions, including altering weather patterns and affecting land-based heatwaves in adjacent regions^16,17^. The 2023 summertime marine heatwave in the North Atlantic was associated with extreme heatwaves over large areas of Europe, particularly during July, and was so large in magnitude that it was a substantial contributor to record global mean temperatures that developed that year (ref. ^18^). There were also severe storms and flooding rains across parts of Europe during June–September, probably exacerbated by enhanced evaporation and higher atmospheric moisture content upstream over the hotter-than-average North Atlantic^19^. Year-long precipitation anomalies for 2023 reveal many regions across Europe and the United Kingdom with total rainfall in the top 10–20% compared with 1991–2020 (ref. ^18^). Given the potential for North Atlantic marine heatwaves to cause substantial ecosystem damage and bring about severe heatwaves and flooding rains over parts of Europe and other regions, it is important to understand the drivers of the extraordinary 2023 event.

## The 2023 North Atlantic marine heatwave

**Fig. 1:**
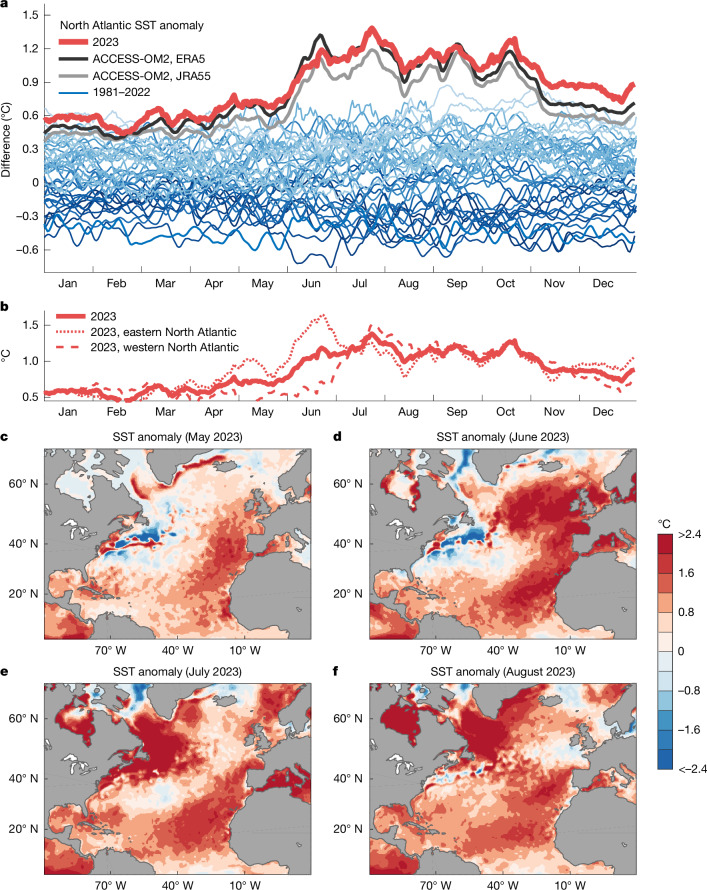
North Atlantic SST anomalies relative to 1981–2010. **a**, Observed daily mean SST anomalies (°C) averaged between the equator and 60° N, with years indicated by colour shaded lines, from dark blues (earliest years) to pale blues (most recent years) and 2023 indicated in bold red. Observed estimates of SST are taken from ERA5. Simulated SST anomalies during 2023 are shown for the model runs forced by ERA5 and JRA55 in black and grey curves, respectively. **b**, Decomposition of total North Atlantic SST anomalies into eastern and western components. The thin red dotted and dashed lines indicate daily averaged SST anomalies for 2023 averaged separately east and west of 40° W, respectively. **c**–**f**, Observed estimates of monthly average SST anomalies during May–August 2023, relative to monthly means calculated over the period 1981–2010.

An analysis of daily mean North Atlantic sea surface temperature (SST) anomalies during 2023 relative to previous years over the satellite measurement era is presented in Fig. 1a. During the Northern Hemisphere summer of 2023, the North Atlantic upper ocean developed exceptionally strong warming, with SST spiking well above previous record temperatures during June and July and remaining well above record levels for the remainder of the year. This temperature evolution is reproduced by the ocean models we later analyse in this study (Fig. 1a). A breakdown of the total 2023 North Atlantic warming into separate western and eastern contributions reveals considerable geographic variations in surface heating (dashed red lines in Fig. 1b), with rapid anomalous warming observed in the eastern North Atlantic during June, followed by a surge in western North Atlantic temperature anomalies during July.

The geographic distribution of anomalous ocean warming during May–August 2023 (Fig. 1c–f) shows that, although initial modest warming west of Europe and North Africa occurred in May, even greater rates of anomalous warming occurred during June, covering much of the eastern North Atlantic with record warm temperatures, at more than 2 °C above average relative to 1981–2010. During July, some of this northeast Atlantic warming abated, whereas—at the same time—the northwestern region flared up with exceptionally warm SST anomalies, extending from the North American coast around Nova Scotia and into the Labrador Sea. Further warming of the oceans adjacent to Greenland and in Hudson Bay occurred during August.

**Fig. 2:**
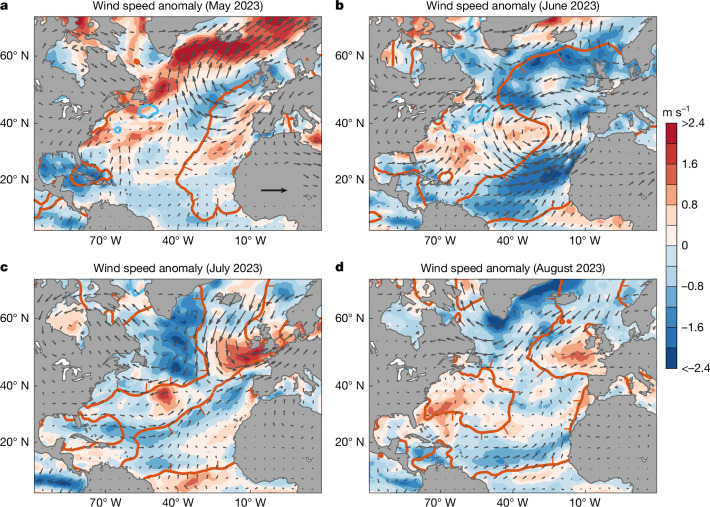
Monthly average surface wind speed and wind vector anomalies during May, June, July and August 2023. **a**–**d**, Wind speed anomalies (m s^−1^) are shaded and calculated relative to the 1981–2010 mean. Wind vector anomaly scale is shown in panel **a**, 5 m s^−1^. Observed estimates of wind speed and wind vectors are taken from ERA5. The +1 °C and −1 °C SST anomaly contours from Fig. 1 are overlaid in red and blue, respectively. To avoid ambiguity, the regions of anomalous warm SST are indicated by the small red tick marks added to the inner side of the +1 °C anomaly contour.

**Extended Data Fig. 1:**
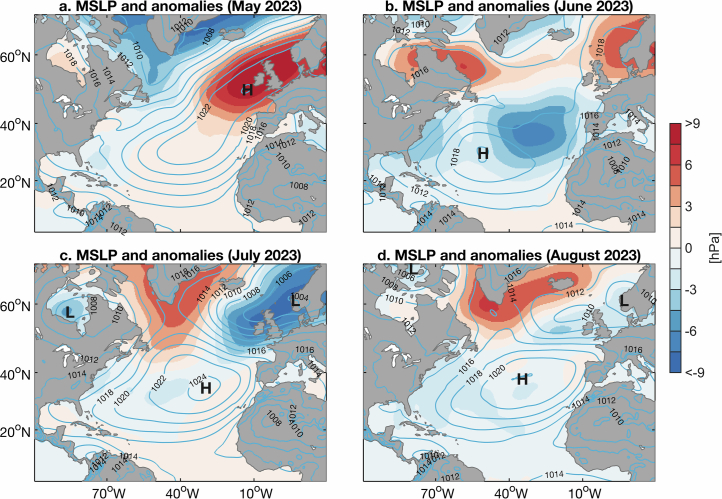
Monthly average sea-level pressure climatology and anomalies during May, June, July and August 2023. **a**–**d**, The mean sea-level pressure (hPa) is shown as contours and anomalies are shaded and calculated relative to the 1981–2010 mean. Mean and anomalous sea-level pressure fields are calculated from hourly ERA5 data.

**Extended Data Fig. 2:**
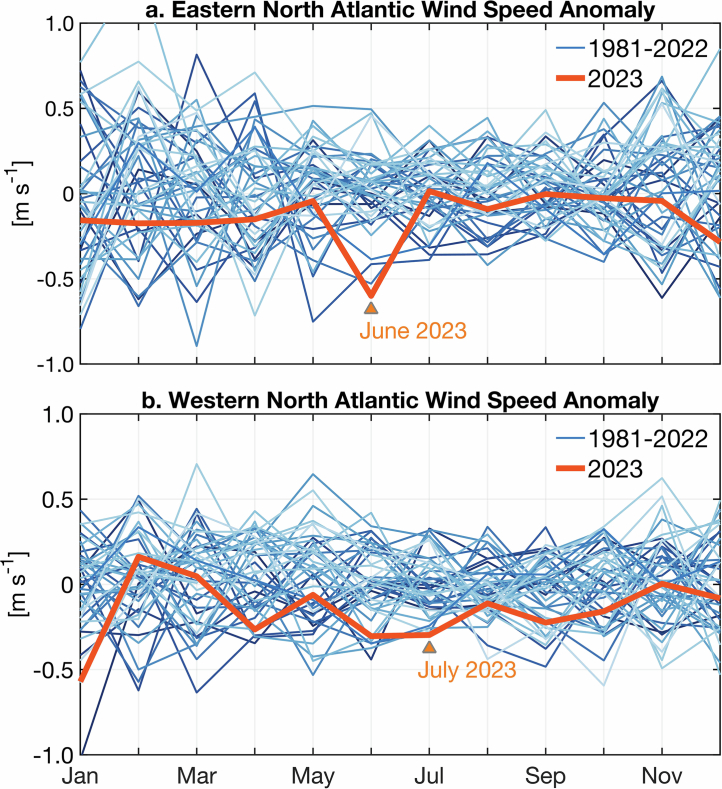
Time series of North Atlantic area-averaged and monthly-averaged wind speed anomalies. Values are calculated between the equator and 60° N, with years indicated by colour shaded lines, from dark blues (earliest years) to pale blues (most recent years) and 2023 indicated in bold red. Units are m s^−1^. Area averages are calculated separately east and west of 40° W. **a**, Eastern North Atlantic. **b**, Western North Atlantic. Wind speeds shown are calculated from hourly ERA5 data.

An examination of the atmospheric circulation and surface wind speed anomalies (Fig. 2) reveals a coincidence between regions of anomalously weak winds and the regions of anomalous North Atlantic surface warming during June and July described above. The anomalously weak winds during June 2023 were largely associated with a weakening of the climatological high-pressure system over the North Atlantic (Extended Data Fig. 1). This weakening of the prevailing anticyclonic circulation is consistent with previously identified teleconnections from a developing El Niño–Southern Oscillation and warmer waters in the tropical eastern Pacific, which act to reduce the northeast trade winds in the Atlantic^20,21^. Further climate or synoptic-scale anomalies probably also played a role, given that the exceptional wind reduction peaked only in June (Extended Data Fig. 2a). During July 2023, weakened winds then became more pronounced in the northwest Atlantic (Fig. 2c and Extended Data Fig. 2b), coinciding with that region’s surge in SST anomalies (compare Fig. 1e with Fig. 1d), whereas to the east, the weakened wind field contracted. At the same time, an anomalous air flow from the north led to a region of SST cooling west of the British Isles. During August, virtually all of the regions that remained anomalously warm coincided with weaker than average winds (Fig. 2d). Relative to the satellite era since 1979, the area-averaged wind speed over the eastern North Atlantic was the lowest ever recorded for June during 2023 and, for the western North Atlantic, the joint lowest ever recorded for July during 2023 (Extended Data Fig. 2), coinciding with the largest surges in SST anomalies seen in Fig. 1b.

**Extended Data Fig. 3:**
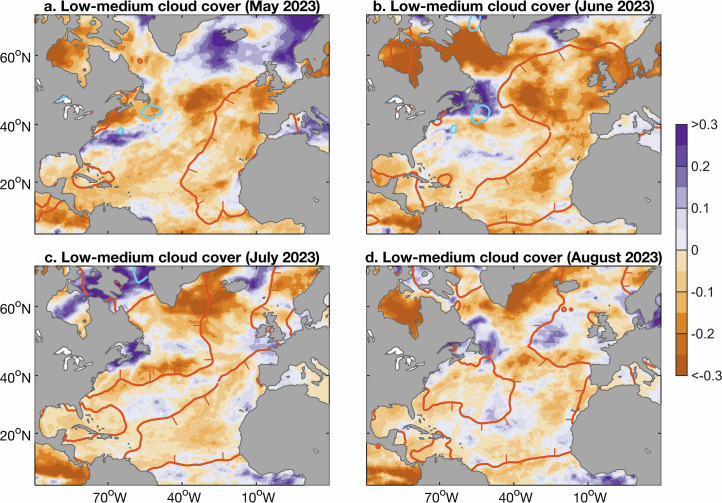
Monthly average low–medium level cloud cover anomalies during May, June, July and August 2023. **a**–**d**, Cloud cover anomalies are shaded and calculated as a fraction (values ranging from 0 to 1) relative to the 1981–2010 mean. Low–medium cloud cover corresponds to the total cloud cover from the surface up to a height of approximately 450 hPa. The +1 °C and −1 °C SST anomaly contours from Fig. 2 are overlaid in red and blue, respectively. To avoid any ambiguity, the regions of anomalous warming are indicated by the small red tick marks added around the +1 °C anomaly contour. Cloud cover anomalies are calculated from hourly ERA5 data.

Reduced winds over the ocean affect air–sea heat fluxes in several ways. First, both latent and sensible ocean heat loss typically decrease, as these exchanges of heat scale with the square of surface wind speed^22^. Reduced ocean evaporation can, in turn, contribute to reduced low-level and mid-level cloudiness, which was indeed the case over regions of low wind speed during June and July 2023 (Extended Data Fig. 3). Furthermore, weakened winds over the ocean may lead to reduced surface albedo as a result of reduced sea-spray aerosols and reduced white-capping from breaking waves^23,24^, which allows more incoming solar radiation to enter the ocean. In combination with clearer skies, low wind speeds are thus conducive to an increase in the net surface heat flux entering the ocean. Once the surface layer of the ocean has warmed, this tends to reduce atmospheric boundary layer stability and increase mixing of dry air at low levels, reducing the low-level relative humidity and leading to fewer low-level clouds. In summer, this leads to an amplifying feedback with further surface ocean warming.

**Extended Data Fig. 4:**
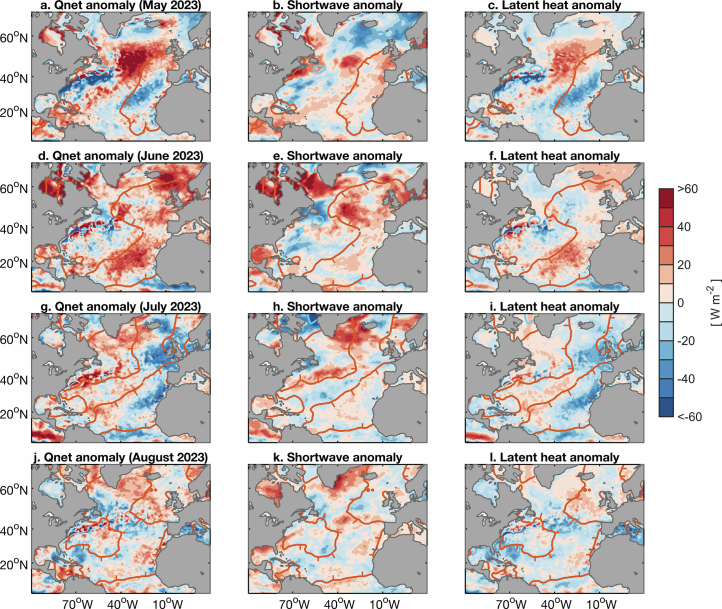
Monthly averaged surface heat flux anomalies during May, June, July and August 2023. **a**–**l**, Shown are the monthly anomalies in the incoming air–sea net surface heat flux *Q*_net_ (left column), the incoming shortwave radiation (middle column) and the latent heat flux (right column). Heat flux anomalies (W m^−2^) are shaded and calculated relative to the 1981–2010 mean. Positive (red) values indicate anomalous heat flux into the ocean, negative values are anomalous ocean heating of the atmosphere. The +1 °C and −1 °C SST anomaly contours from Fig. 2 are overlaid in red and blue, respectively. To avoid ambiguity, the regions of anomalous warming are indicated by the small red tick marks added around the +1 °C anomaly contour. All surface heat flux anomalies are calculated from hourly ERA5 data.

The observed net surface heat flux anomalies during summer 2023 as well as the anomalies in the two dominant components—incoming shortwave radiation and outgoing latent heat fluxes—are shown in Extended Data Fig. 4. This breakdown into constituent terms reveals that the regions of anomalous net ocean heat gain are primarily associated with above-average incoming solar radiation (Extended Data Fig. 4, middle column), with an extra contribution from more localized regions of lower-than-average latent heat loss (Extended Data Fig. 4, right column). Variations resulting from outgoing longwave and sensible heat fluxes are much smaller, with anomalies generally less than about 10 W m^−2^ for most regions (figure not shown). Overall, the regions of greatest warming during June in the eastern North Atlantic and July in the western North Atlantic correspond to regions of anomalous surface ocean heat gain (Extended Data Fig. 4). By contrast, there is anomalous ocean heat loss across much of the eastern North Atlantic during July (Extended Data Fig. 4g), which has been linked to a warming of the atmosphere adjacent to western Europe that contributed to the hot temperatures and heatwaves seen across the region during July^19^. These enhanced evaporative fluxes might also have contributed to the anomalous rainfall that occurred over Europe during July^19^.

As well as the air–sea heat exchange processes described above, the weakened northeasterly trade winds extended over the Sahara Desert, particularly during June 2023 (Fig. 2b). Weakened northeast trades have previously been linked to a reduction in dust transport westward into the Atlantic Ocean^25^. Such a reduction in Saharan dust transport has been associated with warm SST anomalies because dust acts to scatter incoming solar radiation before it reaches the ocean surface^26^. Another factor proposed to account for the increase in incoming solar radiation in 2023 is reduced sulfate aerosol emissions along the main shipping lanes following the International Maritime Organization 2020 agreement^27,28^. However, recent work has suggested that this signal may be difficult to detect on a basin scale given the large interannual variability in Earth’s energy imbalance and the short observational record since these changes came into effect^29^. Although this topic remains an area of continued research, we will later show that, in terms of the record-breaking basin-wide warming of the surface North Atlantic, the relative contribution of solar radiation anomalies was less than the impact of shoaling mixed layers.

**Fig. 3:**
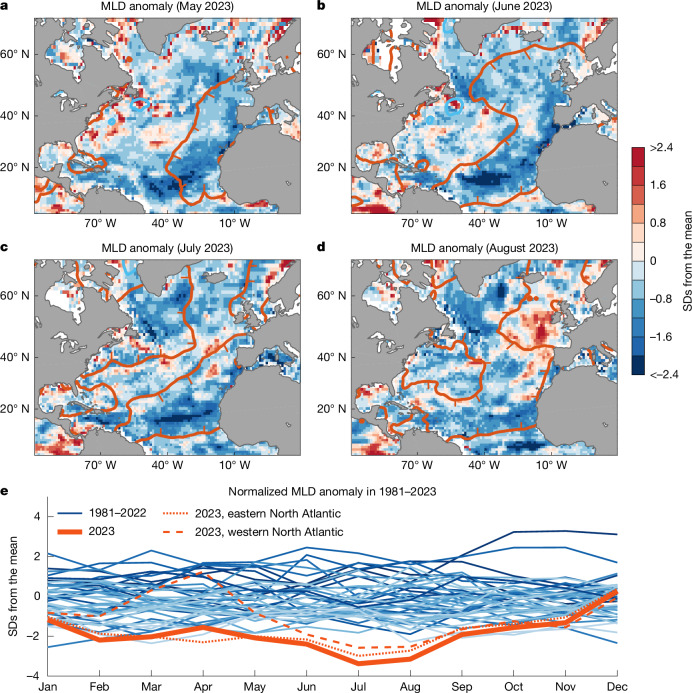
Observed monthly average surface MLD anomalies during May, June, July and August 2023. **a**–**d**, MLD anomalies (shaded) are normalized by one standard deviation (SD) for each month and location and calculated relative to the 1981–2010 mean. The MLD is calculated using a density threshold criterion of 0.125 kg m^−3^, relative to surface density, using temperature and salinity data from the IAPv3 climatology (Methods). The ±1 °C SST anomaly contours from Fig. 2 are overlaid in red and blue, respectively. **e**, Time series of North Atlantic area-averaged MLD anomalies (equator to 60° N), with years indicated by colour shaded lines, from dark blues (earliest years) to pale blues (most recent years) and 2023 indicated in bold red. The thin red dotted and dashed lines indicate monthly averaged MLD anomalies for 2023, averaged separately east and west of 40° W, respectively.

**Extended Data Fig. 5:**
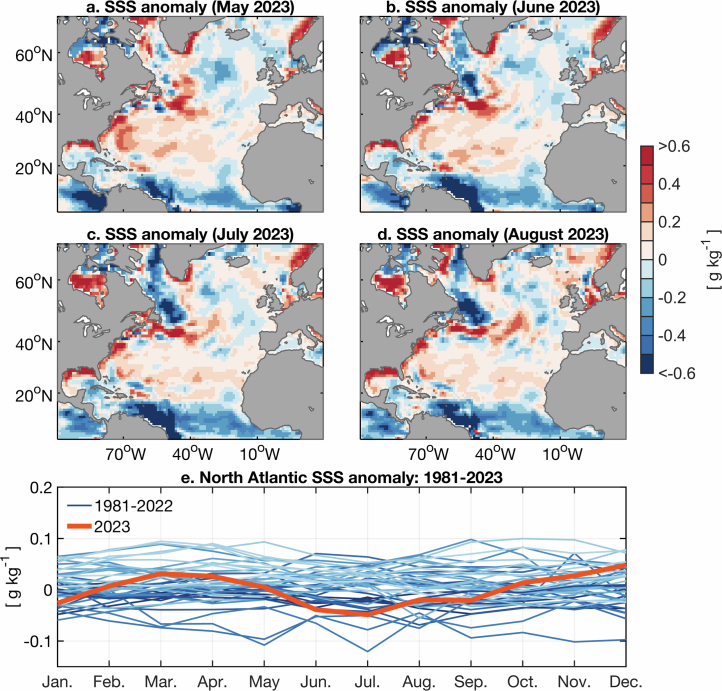
Monthly average surface salinity anomalies during May, June, July and August 2023. **a**–**d**, Salinity anomalies (shaded) are calculated relative to the 1981–2010 mean using monthly IAPv3 data (Methods). **e**, Time series of North Atlantic area-averaged salinity anomalies (equator to 60° N), with years indicated by colour shaded lines, from dark blues (earliest years) to pale blues (most recent years) and 2023 indicated in bold red.

**Extended Data Fig. 6:**
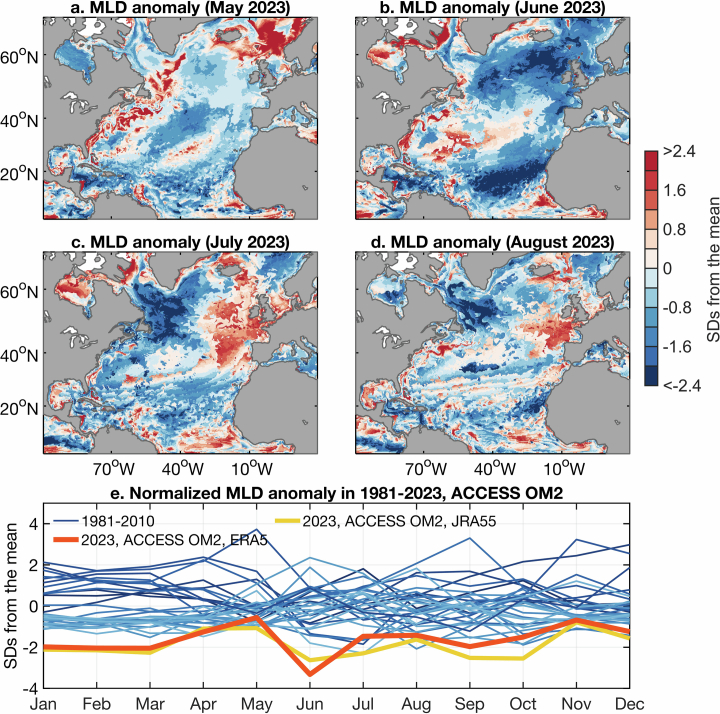
Monthly average model-simulated surface MLD anomalies during May, June, July and August 2023. The model data are taken from the run forced by ERA5 atmospheric variables. **a**–**d**, MLD anomalies (shaded) normalized by one standard deviation (SD) for each month and location, and shown as the number of standard deviations from the 1981–2010 mean. The MLD is calculated using a density threshold criterion of 0.125 kg m^−3^, relative to surface density. **e**, Time series of North Atlantic area-averaged model MLD anomalies (equator to 60° N), with years indicated by colour shaded lines, from dark blues (earliest years) to pale blues (most recent years) and 2023 indicated in bold red. For comparison, the 2023 MLD anomalies in the JRA55-forced model run are also included in panel **e**.

During summer, the surface layer of the ocean becomes well stratified, with warm surface waters overlying colder deep waters. Mixed layer depth (MLD) during this season is thus largely set by the strength of surface winds, which act to mix and overturn surface waters within the wind-driven turbulent mixed layer. It is little surprise then that the regions of record-low wind speeds during summer 2023 largely coincided with unusually shallow mixed layers (Figs. 2 and 3; other ocean reanalysis products are compared in Extended Data Fig. 10). In particular, during June 2023, anomalously shallow MLDs extended from the tropical North Atlantic north to regions west of Europe, with basin averaged MLD anomalies reaching around three standard deviations shallower than average (Fig. 3e; that is, a 3*σ* event, with a likelihood of occurrence of only 0.13% assuming that the MLD anomalies are normally distributed about the mean). Shallow mixed layers can then be seen over the northwest Atlantic during July 2023, coinciding with a region of weaker-than-average winds (Fig. 2c). Part of this region may have also experienced further mixed layer shoaling owing to meltwater input from the nearby Greenland ice sheet (Extended Data Fig. 5), with 2023 being the third highest melt season since passive microwave records began 45 years ago^30^. Melt was particularly high in the southwest of Greenland, with added freshwater entering the Labrador Sea during summer 2023 (ref. ^30^). This region of added meltwater coincided with strong freshening and warming, illustrating that the impact of meltwater on the ocean is not primarily through its temperature but through the dynamical effects of its low salinity and, therefore, low density (and shallow mixed layers). An analysis of the ERA5-forced model-simulated MLD anomalies reveals an overall consistent pattern of record MLD shoaling across the North Atlantic during the summer months of 2023 (Extended Data Fig. 6).

**Extended Data Fig. 7:**
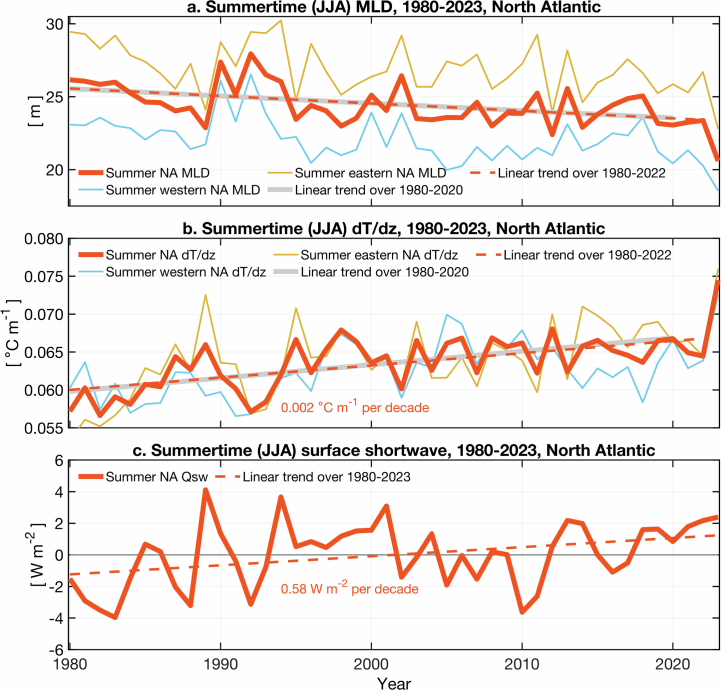
Time series of summertime basin-averaged MLD, upper ocean temperature stratification and incoming solar radiation anomalies. Time series of summertime North Atlantic basin-averaged MLD (m) (**a**), upper ocean temperature stratification (°C m^−1^) (**b**), and incoming solar radiation anomalies (W m^−2^) (**c**). The ocean time series (**a**,**b**) are derived from IAPv3 and the solar radiation anomalies (**c**) from ERA5 (Methods). The upper ocean temperature stratification is calculated between the surface and 50-m depth. All basin-averaged trend lines shown are significant at the 95% confidence level. In panel **a**, the shoaling trend in MLD averaged over June–August is −0.58 m per decade during 1980–2023. In panels **a** and **b**, trend lines are shown excluding and including the period 2020–2022 (after the International Maritime Organization 2020 agreement^27,28^). Time series in panels **a** and **b** are shown for the whole North Atlantic as well as separated into the western and eastern sectors (separated at 40° W).

**Extended Data Fig. 8:**
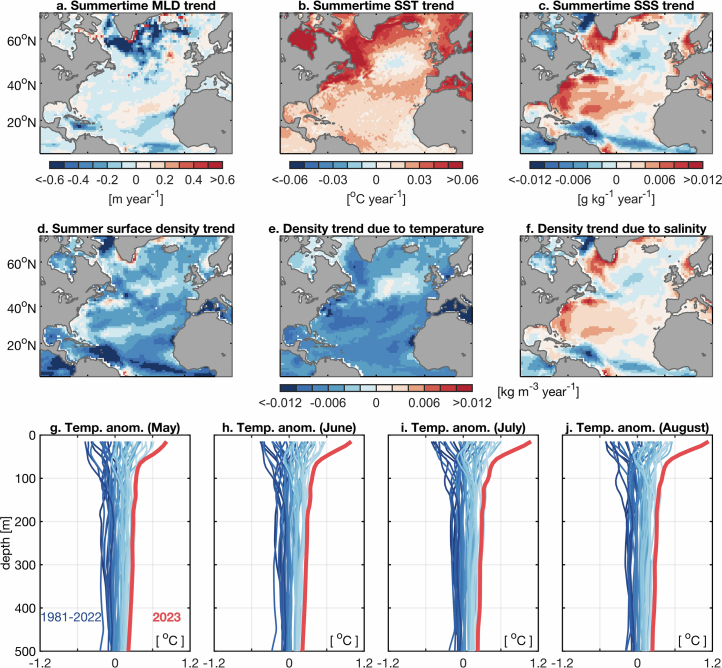
Trends in summertime mean MLD, SST, salinity and density, and anomalies in temperature profiles over the North Atlantic during 1981–2023. Trends are calculated over the period 1981–2023 for the summer June–August season. **a**–**c**, June–August trends in MLD, SST and sea surface salinity (SSS). Units are m, °C and g kg^−1^ per year, respectively. MLD is calculated as the depth at which the monthly averaged potential density in the water column first exceeds the density at 10-m depth by 0.125 kg m^−3^. **d**–**f**, Trends in June–August surface density and the estimated density trend due to surface temperature and salinity effects alone. Units are kg m^−3^ per year. **g**–**j**, Anomalies in temperature profiles averaged over the North Atlantic (equator to 60° N) during May–August 1981–2023, calculated relative to the 1981–2010 climatological mean, with years indicated by colour shaded lines, from dark blues (earliest years) to pale blues (most recent years) and 2023 indicated in bold red. Data for SST are taken from ERA5, whereas surface salinity, density, MLD and subsurface *T*–*S* data are taken from IAPv3 (Methods).

A monthly analysis of observed MLD in the IAPv3 dataset over the past four decades (Fig. 3e) shows a progressive shoaling of mixed layers during summer, with the 2023 MLD anomalies substantially shallower than that expected from the long-term trends alone (Extended Data Fig. 7a). This can also be seen when examining basin-averaged upper ocean temperature stratification and annual mean temperature profiles observed since 1980 (Extended Data Figs. 7b and 8). The IAPv3 shoaling trend averaged over June–August is −0.58 m per decade during 1980–2023, with average North Atlantic MLD in the summer of 2023 (20.6 m) more than 7 m shallower than the peak summer MLD value of 27.9 m observed in the early 1990s (Extended Data Fig. 7a) and around 20% shallower than the climatological summer mean during 1980–2000 (25.1 m). This corresponds to a marked shoaling of the summer mixed layer, reducing the thermal inertia of the surface ocean. Averaged over the North Atlantic, ocean mixed layers were around four standard deviations shallower than average during July 2023 (a 4*σ* event, with a likelihood of occurrence of just 0.003% assuming normally distributed MLD anomalies). During a season of net ocean heat gain, these shallow mixed layers readily warm because less incoming energy is needed to heat a thin surface layer. This triggers an amplifying feedback in which the original ocean warming and stratification, left undisturbed by weak surface winds, leads to further warming and higher levels of stratification (and even shallower mixed layers). A very rapid warming set in as a consequence during June and July 2023.

## Surface temperature budget

To quantify the relative importance of the various driving factors, we next examine a temperature budget of the surface mixed layer during the peak warming months using an ocean model simulation forced by atmospheric reanalysis fields. Two model simulations were evaluated (Methods), with both capturing the main features of North Atlantic anomalous warming during summer 2023 (Fig. 1a). Here we analyse the ocean model forced by ERA5 atmospheric reanalysis fields; similar results are obtained from the JRA55-forced simulation (figures not shown). Observational estimates of the mixed layer temperature (MLT) budget can also be reconstructed, including all components of the surface air–sea heat flux terms, but data are unavailable to resolve the ocean advection and mixing terms, and larger uncertainty exists owing to the sparse measurement record (Methods). Nonetheless, both the observationally derived estimate and the two model simulations show overall agreement in the results obtained from the MLT budget calculations described below.

**Fig. 4:**
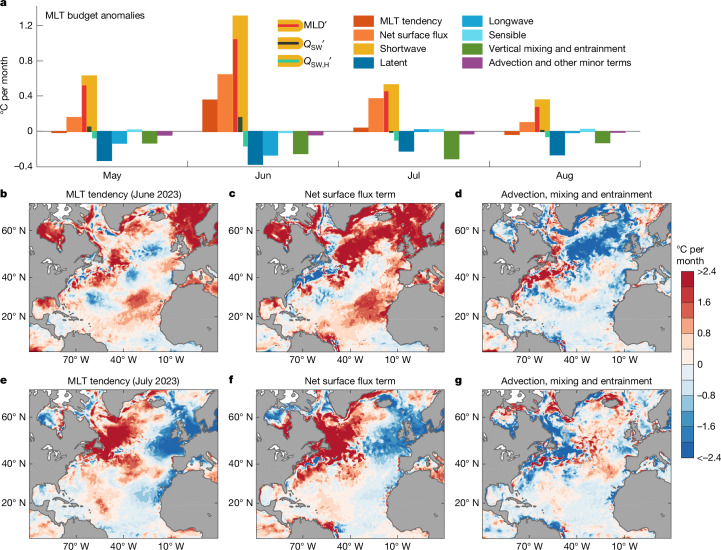
Model-derived North Atlantic surface MLT budget anomalies during 2023 relative to the 1981–2010 climatological mean. **a**, Anomalous surface warming over the North Atlantic (equator to 60° N) during May-August decomposed into each surface heat flux term (and the net surface heat flux), the net vertical mixing and entrainment terms and the advection plus other minor terms. The total anomalous MLT tendency (red bars) is primarily a balance between net surface heat flux anomalies (orange bars) and cooling anomalies at the base of the mixed layer due to vertical mixing and entrainment (green bars). The shortwave radiation anomalies are decomposed into anomalies due to MLD variations alone (MLD′), anomalies due to surface heat flux variations alone (*Q*_SW_′) and anomalies due to the radiative flux through the base of the mixed layer (*Q*_SW,H_′). **b**–**g**, June and July anomalous mixed layer warming (**b**,**e**) versus that due to anomalies in net surface heat fluxes (**c**,**f**) and total ocean advection, mixing and entrainment (**d**,**g**). Units are °C per month throughout. The net surface heat flux anomalies are calculated for the mixed layer, subtracting out shortwave radiation penetration at the base of the mixed layer (Methods). The model simulation shown here is that forced by the ERA5 atmospheric reanalysis fields. Similar results are obtained from the JRA55-forced ocean model simulation. Note that this analysis calculates the MLT budget terms centred on each month, while the warming discernible in Fig. 1c–f is from one month to the next (that is, between monthly averages, centred at the start of each month). Also note that this analysis does not include the effects of interannual variability in shipping emissions. See Methods and Extended Data Fig. 11 for further details.

**Fig. 5:**
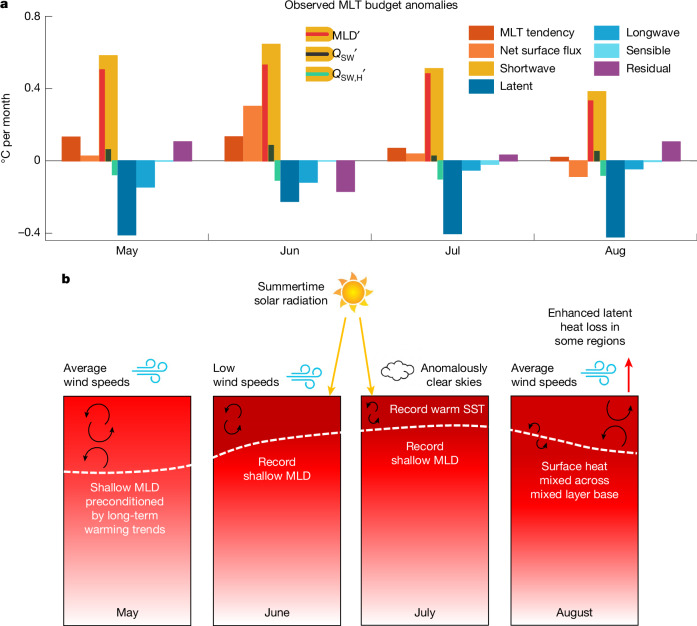
Observed MLT budget anomalies and schematic summarizing the processes that led to the record summer North Atlantic marine heatwave in 2023. **a**, Observed anomalous surface warming over the North Atlantic (equator to 60° N) during May–August, decomposed into each surface heat flux term, with the residual terms including unresolved mixing, entrainment, advection plus uncertainty in the reanalysis estimates. Anomalies are calculated relative to the 1981–2010 climatological mean. The budget terms are calculated using ERA5 air–sea heat fluxes combined with MLD and temperature derived from the IAPv3 climatology (Methods). The shortwave radiation anomalies are decomposed into anomalies due to MLD variations alone (MLD′), anomalies due to surface heat flux variations alone (*Q*_SW_′) and anomalies due to the radiative flux through the base of the mixed layer (*Q*_SW,H_′). The analysis presented here does not include the effects of interannual variability in shipping emissions. See Methods and Extended Data Fig. 11 for further details. **b**, The schematic shows the progression of North Atlantic surface warming during May, June, July and August. Dashed white line indicates the MLD. Shallow mixed layers in May 2023 are part of a long-term trend towards shallower springtime stratification in the North Atlantic. Rapid warming and shoaling of mixed layers in the northeast Atlantic during June was driven by anomalous weak winds and clear skies, leading to much shallower than average mixed layers. That tendency continued during July in the northwest Atlantic. During August, atmospheric winds returned to normal or above-average strength in some regions, leading to anomalous ocean heat loss and atmospheric warming in those areas, as well as anomalous mixing and detrainment of surface layer heat across the base of the mixed layer.

Figure 4a shows an analysis of the ERA5-forced model-simulated total North Atlantic surface warming during May–August 2023, decomposed into anomalies in the MLT tendencies (°C per month), alongside other budget terms relative to the 1981–2010 climatological mean. Note that, because the budget is formulated as a MLT budget, anomalies in each term (for example, the warming due to air–sea heat fluxes) can result from anomalies in the MLD alone or in combination with anomalies in the heat source terms themselves. The geographic distribution of the MLT tendency term is also plotted for June–July 2023 in Fig. 4 (lower panels), separated into anomalies in warming due to net air–sea heat fluxes and anomalies in warming due to the combined effects of ocean circulation and mixing. The corresponding observationally based MLT budget derived from ERA5 air–sea heat fluxes combined with Argo float data and other hydrographic measurements is included in Fig. 5a.

**Extended Data Fig. 9:**
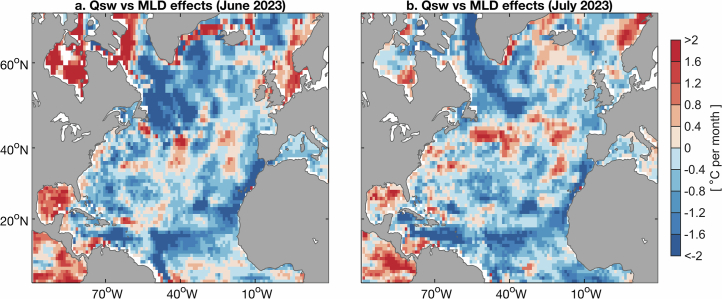
Geographic plot showing locations where anomalously shallow mixed layers (blue regions) or anomalously high incoming shortwave radiation (red regions) dominate the MLT budget. The analysis is shown for the peak warming months of June 2023 (**a**) and July 2023 (**b**), based on observations and reanalysis data. Values are based on the difference between the MLT budget contributions from (1) the net shortwave radiation term (*Q*_SW_) estimated by holding the MLD value at its climatological mean (with *Q*_SW_ varying) and (2) the mixed layer shoaling term estimated by holding incoming shortwave radiation at its climatological mean (with MLD varying). In this way, red shading denotes areas in which the solar radiation effect dominates the MLT anomalies and blue shading denotes regions in which MLD shoaling dominates the MLT anomalies. The budget terms are calculated using ERA5 air–sea heat fluxes combined with MLD and MLT derived from IAPv3 (Methods). The analysis presented here does not include the effects of interannual variability in shipping emissions. See Methods and Extended Data Fig. 11 for further details estimating shipping emission effects.

The model and observed MLT budgets both reveal that the record surface ocean warming was because of a combination of anomalously shallow mixed layers, clear skies and high incoming shortwave radiation (Figs. 4 and 5a). However, a breakdown of the relative roles of anomalous surface heat fluxes versus anomalous shallow MLDs (bars shown in the *Q*_SW_ term; Figs. 4a and 5a) reveals that the exceptionally shallow mixed layers played the dominant role in the basin-wide warming, such that the marine heatwave would have been almost as severe had air–sea heat fluxes been no different to the climatological summer mean. By contrast, solar radiation anomalies had a more localized impact. A regional analysis reveals that the main areas where above-average incoming solar radiation played the dominant role (Extended Data Fig. 9) coincided with reduced low-level to mid-level cloud cover (Extended Data Figs. 3 and 4). Some of these regions also coincide with locations of the main shipping lanes in which reduced sulfate emissions have been linked to clearer skies and higher incoming solar radiation^31–34^. Part of the reduction in low-level cloud cover could thus be linked to indirect effects from reduced shipping emissions^35^. Overall, our regional temperature budget analysis demonstrates that, although the basin-wide surface warming was driven primarily by shoaling mixed layers, reduced shipping emissions could have played a localized role in the 2023 North Atlantic marine heatwave. Uncertainty remains nonetheless in quantifying the direct and indirect radiative impacts of reduced shipping emissions^32,35^, with other factors also potentially contributing to these shortwave radiation anomalies, such as reduced cloud cover due to variations in weather and climate modes, proximity to the solar maximum (reached in 2024), surface albedo effects, and atmospheric water vapour^35^.

The regions that cannot be explained by the air–sea heat flux terms include the Gulf Stream and its extension, where enhanced northward advection of warm tropical surface waters dominates (Fig. 4). Elsewhere, in the regions indicated by blue shading in the model ocean circulation and mixing terms (Fig. 4d,g), the anomalous surface heating exceeds the amount of energy required to account for the surface layer warming. In these regions, there is enhanced vertical heat loss across the base of the summer mixed layer, owing to both shortwave penetration effects as well as anomalous vertical entrainment and mixing (Fig. 4a). This results in substantial heating of the water column below the mixed layer, as can also be seen in the observed temperature–depth anomaly profiles (Extended Data Fig. 8, lower panels). Overall agreement can be seen in the observational MLT budget terms, with shoaling mixed layers the dominant factor contributing to the record-breaking basin-wide marine heatwave, alongside a regional contribution from anomalous incoming shortwave radiation (Fig. 5a and Extended Data Fig. 9).

## Mechanisms driving the extreme heating

A schematic outlining the progression of surface mixed layer warming over the Northern Hemisphere summer in the Atlantic Ocean is shown in Fig. 5b. During June in the east and then subsequently July in the west, record weak winds over the surface of the ocean led to the shallowest ever recorded MLDs in those regions. Coupled with clearer-than-average skies, lower cloud albedo^27^ and net heat gain across the air–sea interface, this led to an amplifying feedback that produced very shallow and very warm mixed layers across much of the North Atlantic Ocean. Although the progression of surface warming played out separately over the eastern North Atlantic during June and then in the northwestern North Atlantic during July, the same overall mechanism seems to have controlled both bursts of surface warming; namely, surface heating driven by anomalously weak winds, clear skies and shallow mixed layers. Reduced sulfate aerosol emissions along the main Atlantic shipping lanes^27,28,31,32^ has also been proposed as a driver of the record-breaking 2023 warming. By recalculating the North Atlantic temperature budget anomalies assuming (1) climatological air–sea heat fluxes and (2) climatological MLDs (Figs. 4a and 5a), we have demonstrated that, at a basin scale, shallow mixed layers resulting from anomalous weak winds were the dominant driver of the record basin-wide North Atlantic warming. We also find evidence that reduced shipping emissions could have played a localized role, with anomalous clear skies contributing enhanced warming anomalies along a band of latitudes coinciding with the region’s main shipping lanes (Extended Data Fig. 9). Given that ERA5 does not include interannual variability in shipping emissions, we further tested these results by recalculating the MLT budget with an additional +1 W m^−2^ anomaly in incoming solar radiation applied everywhere over the North Atlantic (Methods). This shortwave radiation anomaly is approximately double the basin-average estimate for the impact of reduced shipping emissions over the North Atlantic in ref. ^31^, which is at the high end of available estimates. In all analyses shown, both model and observations, our results remain robust to this addition of 1 W m^−2^ solar radiation, with minimal changes to the results shown (see, for example, Extended Data Fig. 11).

An analysis of the past few decades of ocean and atmosphere measurements reveals no notable trend in surface wind speed over the North Atlantic Ocean, but there is a marked trend towards shallower MLDs across all seasons (Fig. 3e and, for summer, Extended Data Figs. 7a, 8a), as well as a notable trend towards increased incoming solar radiation in summer (Extended Data Fig. 7c). In the absence of surface wind trends, the mixed layer shoaling trend must be driven by a combination of stronger summer heating and surface freshwater input, both of which act to enhance the stratification of the upper ocean. This is analysed in Extended Data Fig. 8b,c, which shows the 1981–2023 multidecadal trends in North Atlantic SST and sea surface salinity. Also shown in Extended Data Fig. 8d–f are trends in summertime sea surface density, alongside the estimated density trend owing to surface temperature and salinity effects alone. This analysis reveals that the shoaling North Atlantic mixed layer trend over the past four decades is primarily driven by surface ocean warming, with a smaller contribution from surface freshening in the tropics and subpolar latitudes. The multidecadal surface ocean warming is primarily because of long-term anthropogenic warming, with a possible further contribution from a trend in incoming solar radiation (estimated to be +0.58 W m^−2^ per decade from ERA5 data; Extended Data Fig. 7c), which is consistent with reduced shipping emissions and variations in the solar cycle, as noted in previous work^35^. Overall, warming and freshening has led to much shallower MLDs in the past decade compared with the late twentieth century. A trend towards shallower mixed layers preconditions the Atlantic for an increase in the likelihood of severe summertime marine heatwaves of the type observed in 2023. Similar preconditioning of severe marine heatwaves has also been noted for the North Pacific Ocean^36,37^.

## Summary and conclusions

To summarize, the anomalous warming of the surface mixed layer in the North Atlantic Ocean during summer 2023 was unprecedented in both spatial scale and magnitude over the observational record, forming a near-basin-wide anomaly that has persisted over much of the basin until today. We identify the dominant drivers of this extreme North Atlantic marine heatwave to be low wind speeds and anomalously shallow mixed layers, alongside a regional contribution from anomalous clear skies and increased surface ocean heat flux. Moreover, our study highlights the importance of a shoaling mixed layer in the development of extreme summertime marine heatwaves, because during seasons of climatological warming, a shallow mixed layer can lead to rapid surface warming without the need for anomalous radiation or turbulent heat fluxes into the ocean.

The record shallow mixed layers seen in 2023 seem to have been bolstered by a long-term mixed layer shoaling trend observed in the North Atlantic, one that is projected to continue into the future. The likelihood of similar events occurring is thus expected to increase in the coming years and beyond. With the prevailing atmospheric circulation moving oceanic air masses towards Europe and other regions, alongside the proximity of the summertime heating to Greenland and the source waters for the Atlantic meridional overturning circulation, this has potential major implications for future climate at regional to global scales.

## Methods

### Observational and reanalysis data

The atmospheric and air–sea flux fields analysed in this study are taken from the European Centre for Medium-Range Weather Forecasts (ECMWF) Reanalysis Version 5 (ERA5)^38^, using daily mean fields derived from hourly data and monthly averaged fields from 1981 to 2023 over a 1/4° horizontal resolution grid. Variables analysed from ERA5 include surface wind speed (m s^−1^) and surface wind vectors, surface air–sea heat fluxes (W m^−2^), mean sea-level pressure (hPa) and cloud cover fraction. SST (°C) is also taken from ERA5. The net air–sea heat flux is the sum of incoming shortwave radiation less outgoing latent, sensible and longwave radiative heat fluxes.

The surface net shortwave (solar) radiation corresponds to the amount of solar radiation that reaches the surface of the Earth (both direct and diffuse) minus the amount reflected by the Earth’s surface (which is governed by the albedo). In the ERA5 reanalysis, only climatological aerosol effects and aerosol changes due to large volcanic eruptions are incorporated into the estimate of the net surface shortwave (solar) radiation. In particular, the incoming solar radiation is partly reflected back to space because of both clouds as well as particles in the atmosphere (aerosols), but without interannual variability in aerosol effects aside from volcanic eruptions. The remainder is incident on the Earth’s surface, for which some of it is reflected owing to surface albedo effects. ERA5 assimilates cloud liquid water from a variety of satellites, as detailed in the ERA5 online documentation, formulating cloud fraction and cloud albedo as surface and single-level parameters of the reanalysis. To test the sensitivity of our results to aerosol effects from reduced shipping emissions, we further analysed the observed and modelled MLT budgets by adding 1 W m^−2^ to the solar radiation terms for all months in 2023, and also to just the summer months May–August when solar radiation effects are at their maximum. The added heat flux of +1 W m^−2^ is approximately double the basin-average estimate for the impact of reduced shipping emissions over the North Atlantic of 0.56 W m^−2^ in ref. ^31^, which is at the high end of available estimates. In all cases our results remain robust to this addition of 1 W m^−2^ solar radiation, with negligible changes to the results shown (Extended Data Fig. 11).

Subsurface ocean temperature and MLDs are obtained from an observation-based dataset; the Institute of Atmospheric Physics (IAP) hydrographic data^39–41^ version 3 available from 1981 to 2023 with 1° horizontal resolution (hereafter referred to as ‘IAP data’). The main latitude band of interest spans the North Atlantic from the equator to 60° N, which mostly avoids regions of low data coverage, such as the high-latitude regions, particularly ice-covered areas. Sparse coverage by Argo floats is, however, a problem in marginal seas, although the gridded IAP climatology merges both Argo and other historical hydrographic observations, including XBTs. Nonetheless, reliable reconstruction of interior ocean temperature and MLD is only available at monthly mean resolution. The Gibbs SeaWater (GSW) Oceanographic Toolbox^42^ from the TEOS-10 software^43^ is applied to convert the IAP in situ temperature to conservative temperature^44^. Static stability of the water column on timescales of months is achieved by applying vertical stabilization software^45^. The surface-referenced potential temperature, *θ*, and potential density, *ρ*, are then calculated using functions from the GSW Oceanographic Toolbox.

Ocean MLD is calculated as the depth at which the monthly averaged potential density in the water column first exceeds the density at 10-m depth by 0.125 kg m^−3^, following previous work^46^. The MLD anomalies and the related MLT budget analyses were repeated using a density threshold of 0.03 kg m^−3^ and the results presented here are robust to this choice. MLD anomalies were also re-evaluated using a reference depth of 5 m instead of 10 m and robust patterns were obtained. The separate contributions of *T* and *S* to the surface density trends were also calculated (Extended Data Fig. 8). This was done by combining the monthly climatological salinity field (averaged during 1981–2010) with the time-varying temperature fields to derive the temperature contribution. When estimating the salinity effects on density trends, the opposite was done, namely, using the climatological mean temperature fields alongside the time-varying salinity fields.

**Extended Data Fig. 10:**
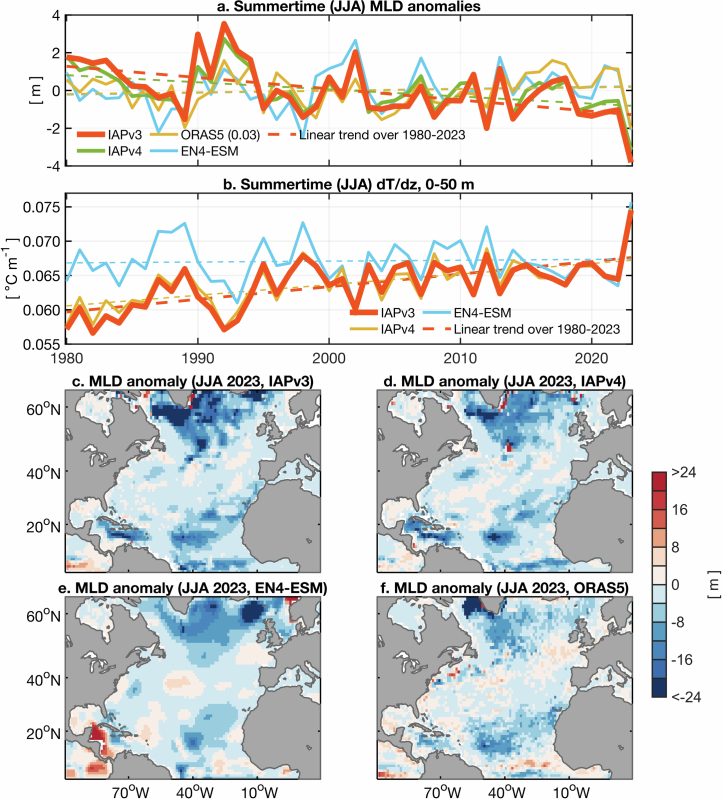
Anomalies in summertime North Atlantic MLD and vertical gradients in temperature using different ocean reanalysis products. **a**, Time series of summertime (June–August) basin-averaged North Atlantic MLD (m) anomalies during 1980–2023. **b**, Same as **a** but showing basin-averaged d*T*/d*z* (°C m^−1^). **c**–**f**, Summertime anomalies in MLD (m) estimated during 2023 from the IAPv3, IAPv4, ORAS5 and EN4-ESM reanalysis products. Linear trends are indicated by the dashed lines in panels **a** and **b**.

**Extended Data Fig. 11:**
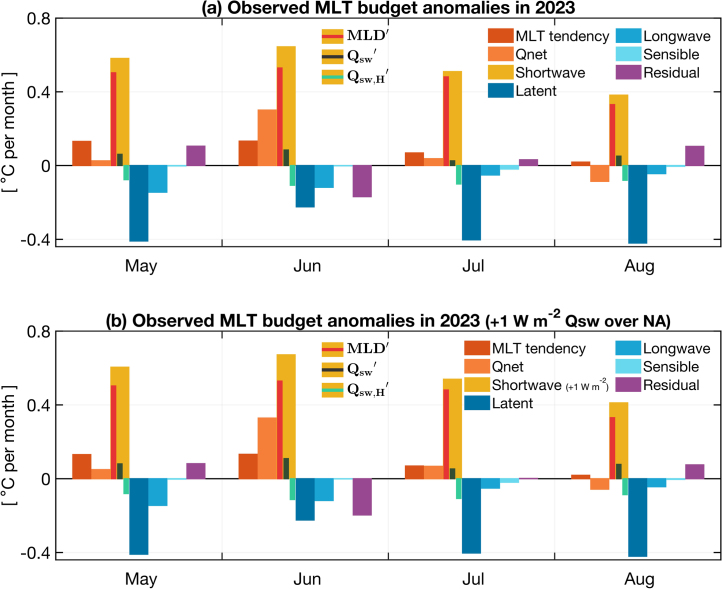
Analysis of potential shipping emissions impacts on the observed mixed layer temperature budget during the 2023 North Atlantic marine heatwave. **(a)** MLT budget as per Fig. 5a of the main paper, included here for comparison, using ERA5 estimates for the surface heat flux terms. Note that the ERA5 reanalysis does not include the effects of interannual variability in shipping emissions. **(b)** Same as panel **(a)**, but showing the MLT budget terms recalculated imposing an additional 1 W m^−2^ anomaly in the incoming solar radiation term *Q*_SW_ during 2023, to examine the potential impact of reduced shipping emissions on the observed MLT budget. The additional 1 W m^−2^ is applied over the whole North Atlantic in 2023. For reference, estimates of the impact of reduced shipping emissions typically vary between 0.1 — 0.3 W m^−2^, with highest estimates for the basin-averaged North Atlantic corresponding to an increase in *Q*_SW_ of 0.56 W m^−2^ during 2023 (ref. ^31^). In this way, panel **(b)** can be interpreted as an upper bound on the effects of shipping emission reductions in the MLT budget calculation. The methods and bar chart terms shown match the terms shown in Fig. 5a.

The robustness of the estimated 2023 anomalies in MLD derived from IAPv3 data was evaluated by examining the corresponding MLD anomalies in the IAPv4, ORAS5 and ensemble mean of EN4.2.2 datasets (EN4-ESM). Overall consistent patterns of record shallow MLD anomalies were obtained during 2023 in all four datasets (Extended Data Fig. 10). However, there are variations in the long-term multidecadal trends in MLD and upper ocean temperature gradients estimated by the different products (Extended Data Fig. 10), consistent with previous findings for the North Pacific^36^. The differences in long-term trends are in part the result of data sparsity before the Argo period, although variations can also be seen in the more recent record. Note also that the EN reanalysis is less reliable for long-term trend estimates^47^, as missing values relax to climatology in the absence of any observations. Nonetheless, in all ocean reanalyses considered, 2023 sees a sudden anomalous shoaling of the mixed layer compared with previous years.

### Ocean model simulations

Two ocean model simulations forced by different observation-based atmospheric reanalysis fields, namely, ERA5 and JRA55-do, were integrated to evaluate the driving mechanisms of the North Atlantic peak warming during 2023. The two model simulations both capture the temporal evolution of the onset of surface layer warming over the North Atlantic during 2023, particularly the ERA5-forced simulation (Fig. 1a). The model configurations are taken from the Australian Community Climate and Earth System Simulator Ocean Model Version 2 (ACCESS-OM2) with the configurations run at a nominal 0.25° horizontal resolution with 50 vertical levels (surface grid cell thickness of 2.3 m). ACCESS-OM2 is a global ocean–sea-ice model driven by a prescribed atmosphere; the version used here has been described previously^48^, with improvements described by follow-up work^49^. The ocean model component is the Modular Ocean Model (MOM) version 5.1 (ref. ^50^), coupled to the Los Alamos sea ice model version 5.1.2 (ref. ^51^). Vertical mixing in the boundary layer is parameterized using the *K* profile parameterization (KPP^52^). Further model details can be found in previously published work^48,49^.

The forcing of the two ocean model simulations is as follows. In the main run analysed here, referred to as the ERA5 simulation, the model is forced by ERA5 atmospheric reanalysis fields^38^. In the second simulation, referred to as the JRA55 run, the Japanese 55-year Reanalysis (JRA55-do) dataset for forcing ocean–sea-ice models is used. In the JRA55 run, after spinning up over six cycles forced by the 1958–2018 JRA55-do v1.4 fields (following the OMIP-5 spinup protocol^53^), the sixth cycle is extended using JRA55-do v1.5.0 for 2019 and the delayed-mode JRA55-do v1.5.0.1 to the end of 2023 (ref. ^54^). The ERA5 run is initialized at the end of 1957 and then run over 1958–2023 using ERA5 atmospheric forcing, with runoff taken from JRA55-do (as for the JRA55 run). Both model runs were initialized using World Ocean Atlas 2013 v2 temperature and salinity fields^55,56^.

The model data analysed here include SST and surface heat fluxes from both the ERA5 and JRA55 simulations and, for the ERA5 run, all variables required to reconstruct a surface MLT budget (detailed below) both during 2023 and also during all years of the model simulation from 1981 onwards, to construct the baseline 1981–2010 climatological mean MLT budget from which we evaluate the 2023 anomaly fields. The North Atlantic SST evolution of the two model runs is, overall, consistent with observations (Fig. 1a), as is the pattern of MLD shoaling across the North Atlantic during 2023 (Extended Data Fig. 6). The magnitude of air–sea surface heat flux anomalies during 2023 is also consistent with observations, as revealed in the breakdown of the anomalous net surface heat flux contributions to the MLT budget during May–August (compare Figs. 4a and 5a). The geographic pattern of net surface-heat-flux-driven MLT warming also compares well between both model runs and observations.

### Surface layer temperature budget

A budget for the upper ocean MLT can be written as

$$\frac{\partial {\theta }_{{\rm{h}}}}{\partial t}=\frac{{Q}_{{\rm{net}}}-{Q}_{{\rm{h}}}}{{\rho }_{0}{c}_{{\rm{p}}}h}+{\rm{advection}}+{\rm{entrainment}}+{\rm{mixing}}$$

in which *θ*_h_ is the MLT, *t* is time, *Q*_net_ is the net surface air–sea heat flux (W m^−2^), *Q*_h_ is the heat flux corresponding to shortwave radiation penetration across the base of the mixed layer (also in W m^−2^), *ρ*_0_ is seawater density (taken to be 1,027 kg m^−3^), *c*_p_ = 3,992 J K^−1^ kg^−1^ is the heat capacity of seawater, *h* is the MLD (m), advection represents net heating owing to three-dimensional ocean advection, entrainment represents the heat flux associated with mixed layer shoaling or deepening and the mixing term includes the net heat flux owing to parameterized subgrid-scale processes such as vertical mixing and eddy-induced mixing. The entrainment term can be written as:

$$-\frac{1}{h}\frac{\partial h}{\partial t}({\theta }_{{\rm{h}}}-{\theta }_{{\rm{ent}}}),$$

representing entrainment associated with mixed layer shoaling or deepening, with *θ*_ent_ the temperature of detrained/entrained water and *θ*_h_ the MLT.

The shortwave penetration across the base of the mixed layer (*Q*_h_) in the model is computed online and output as a heat budget diagnostic by MOM5, in which shortwave radiation is distributed into the ocean interior as a function of depth^57^, modulated by the seasonal climatological chlorophyll distribution^58^. The penetration of shortwave radiation across the base of the mixed layer for the observationally based estimate is taken as *Q*_h_ = *Q*_SW_. *F*(*z*), in which *Q*_SW_ is the shortwave radiation across the air–sea interface from the ERA5 reanalysis and *F*(*z*) is the exponential decay function^59^,

$$F(z)=R{{\rm{e}}}^{-\frac{z}{{h}_{1}}}+(1-R){{\rm{e}}}^{-\frac{z}{{h}_{2}}}$$

where *z* is depth (positive downwards), *R* *=* 0.58, *h*_1_ = 0.35 m and *h*_2_ = 23 m.

The two ocean model simulations give, overall, robust results in terms of the magnitude of the anomalous surface heat flux contributions to the MLT tendency terms (figure not shown), including the breakdown into shortwave, longwave, sensible and latent heat flux terms. The focus of our more detailed MLT budget analysis is the ERA5 run, which was integrated with all required terms saved to quantify monthly variations in the MLT balance, including three-dimensional ocean advection and mixing, as described below. The temperature budget of the ERA5 model can also be compared with estimates using the observationally based ERA5 reanalysis combined with IAP estimates of surface ocean temperature and mixed layer fields. However, insufficient observational data are available to constrain the other surface MLT budget terms, such as ocean advection and mixing, as well as vertical entrainment. We can nonetheless compare the surface air–sea heat flux contributions across the model and observed reanalysis fields.

All terms in the model MLT budget, including all components of ocean advection, mixing and entrainment, can be diagnosed by integrating the fully closed cell-by-cell model heat budget^50,60,61^ over the surface mixed layer (defined here using the same definition as the observed MLD, described above) and dividing by the surface MLD. Here this integration is performed using monthly averaged heat budget diagnostics and the monthly averaged MLD. The entrainment term, the only term not explicitly included in the three-dimensional model heat budget, is computed by residual as the difference between the tendency of the MLT (computed by taking the difference of snapshots of the temperature and the MLD at the beginning and end of each month) and the total mixed layer heat content tendency (that is, the sum of all point-by-point heat budget processes, divided by *ρ*_0_*c*_p_*h*). Such a calculation produces a closed budget but neglects correlations between submonthly variations in the MLD and cell-by-cell model heat budget terms. However, a comparison performed in 2023 between these monthly averaged budget terms and similar terms obtained using daily averages (only available for 2023) showed only small differences (order less than 5%; figure not shown).

The MLT budget analysis for observations (Fig. 5) is derived by comparing the estimated mixed layer heat storage rate to the heating and cooling driven by surface heat fluxes alone (less shortwave penetration at the base of the mixed layer), with the residual implicitly including all ocean advection, entrainment and mixing terms, as well as any errors owing to incomplete data coverage in space and time, uncertainty in estimating MLDs and temperatures and uncertainty in the ERA5 net air–sea heat fluxes (both in 2023 and in the baseline period 1981–2010). As for the model simulations, the observational budget also neglects submonthly correlations between the surface heat flux and MLD variations, although analysis of the model budget at daily and monthly timescales suggests that this term is small. The MLT tendency, ∂*θ*_h_/∂*t*, is calculated from monthly observations as a centred second-order difference of monthly mean MLT. With only monthly MLD values available from observations, this results in substantial smoothing of the MLT warming signal compared with the daily resolved model MLT budget.

The pattern of residual terms seen in the observed calculation reveals features that include unresolved temperature changes due to ocean circulation effects both laterally, such as in the Gulf Stream, and also vertically, because of entrainment and vertical mixing at the base of the mixed layer. By contrast, the MLT budget of the model explicitly resolves the contributions from ocean circulation and mixing, and so all terms can be shown.

The shortwave radiation anomalies in the MLT budget are further diagnosed by recalculating these anomalies during 2023 but separately considering the effects on MLT of 2023 anomalies in MLD, surface incoming shortwave radiation and radiation through the base of the mixed layer. This calculation simply recomputes the 2023 MLT budget holding all other variables at their climatological mean, then separately including the 2023 anomalies in MLD (MLD′), surface shortwave heat flux (*Q*_SW_′) and anomalies owing to the radiative flux across the base of the mixed layer (*Q*_SW,H_′). The resulting values are shown from the ERA5-forced model run and observations in Figs. 4a and 5a and Extended Data Fig. 9, respectively. This decomposition indicates the sign and magnitude of the shortwave radiation component of the MLT budget anomalies assuming that only one of these three factors (MLD′, *Q*_SW_′ and *Q*_SW,H_′) varied with its 2023 values. For example, the MLD′ values indicate the size of the 2023 shortwave radiation term assuming that the incoming shortwave radiation, and the radiation through the base of the mixed layer, followed the climatological mean. The *Q*_SW_′ term conversely takes MLD and *Q*_SW,H_ to follow the climatological mean, with *Q*_SW_ varying with its 2023 values. Last, *Q*_SW,H_′ assumes that *Q*_SW_ and MLD follow the climatological mean, with only the radiative flux through the base of the mixed layer evolving with its 2023 values. This approach is a simple way to evaluate what factors were the most important in generating the summertime warming in the North Atlantic during 2023, although the contributions are not additive to unity by definition.

## Online content

Any methods, additional references, Nature Portfolio reporting summaries, source data, extended data, supplementary information, acknowledgements, peer review information; details of author contributions and competing interests; and statements of data and code availability are available at 10.1038/s41586-025-08903-5.

## Data Availability

The ACCESS-OM2 model hindcast simulations analysed in this study are available at the Consortium for Ocean–Sea Ice Modelling in Australia (COSIMA) data collection repository at https://zenodo.org/records/14942468. The ERA5 data analysed in this study are available from https://cds.climate.copernicus.eu/. The Institute of Atmospheric Physics gridded ocean temperature and salinity data analysed in this study are available at http://www.ocean.iap.ac.cn/. The ORAS5 global ocean reanalysis data are available from https://cds.climate.copernicus.eu/. The latest version of the EN4 reanalysis (EN4.2.2) is available from https://www.metoffice.gov.uk/hadobs/en4/download-en4-2-2.html. The ERA5 data used to force the ERA5 ocean model simulations are available from https://cds.climate.copernicus.eu/. The JRA55-do data used to force the JRA55 model simulations are available from https://climate.mri-jma.go.jp/~htsujino/jra55do.html. All base maps shown in this paper were generated using the reanalysis, observational and model grids, which delineate between ocean and land data points.

## Code availability

The ACCESS-OM2 model components are all open source. The ACCESS-OM2 code is available at https://github.com/COSIMA/access-om2/. All analysis code scripts written to generate the figures and diagnostics described in this study are available at the GitHub repository https://github.com/ZhiLiUNSW/North_Atlantic_2023_Marine_Heatwave.
